# Regeneration mechanism for skin and peripheral nerves clarified at the organ and molecular scales

**DOI:** 10.1016/j.cobme.2017.12.002

**Published:** 2018-06

**Authors:** Ioannis V. Yannas, Dimitrios S. Tzeranis, Peter T. C. So

**Affiliations:** a Department of Mechanical Engineering Massachusetts Institute of Technology, Cambridge, MA 02139, USA; b Department of Biological Engineering, Massachusetts Institute of Technology, Cambridge, MA 02139, USA

**Keywords:** Regeneration, Skin, Peripheral nerves, Phenotype change, Contraction blocking, Integrin-ligand interaction

## Abstract

This article is a review of current research on the mechanism of regeneration of skin and peripheral nerves based on use of collagen scaffolds, particularly the dermis regeneration template (DRT), which is widely used clinically. DRT modifies the normal wound healing process, converting it from wound closure by contraction and scar formation to closure by regeneration. DRT achieves this modification by blocking wound contraction, which spontaneously leads to cancellation of scar formation, a process secondary to contraction. Contraction blocking by DRT is the result of a dramatic phenotype change in contractile cells (myofibroblasts, MFB) which follows specific binding of integrins α_1_β_1_ and α_2_β_1_ onto hexapeptide ligands, probably GFOGER and GLOGER, that are naturally present on the surface of collagen fibers in DRT. The methodology of organ regeneration based on use of DRT has been recently extended from traumatized skin to diseased skin. Successful extension of the method to other organs in which wounds heal by contraction is highly likely though not yet attempted. This regenerative paradigm is much more advanced both in basic mechanistic understanding and clinical use than methods based on tissue culture or stem cells. It is also largely free of risk and has shown decisively lower morbidity and lower cost than organ transplantation.

## Introduction

Researchers have spent substantial efforts during the past decades to generate physiologically functioning organs *in vitro*. These efforts have been based on culturing cells of various types, often with added growth factors, and on use of a large variety of biomaterials, typically in the form of gels and scaffolds, but without sufficient success to warrant routine clinical use. In a different approach, stem cell methodology has been advanced as a potentially revolutionary approach that would ideally recapitulate biological development and grow organs thereby; however, dependable clinical translation has not been achieved yet.

**Fig. 1:**
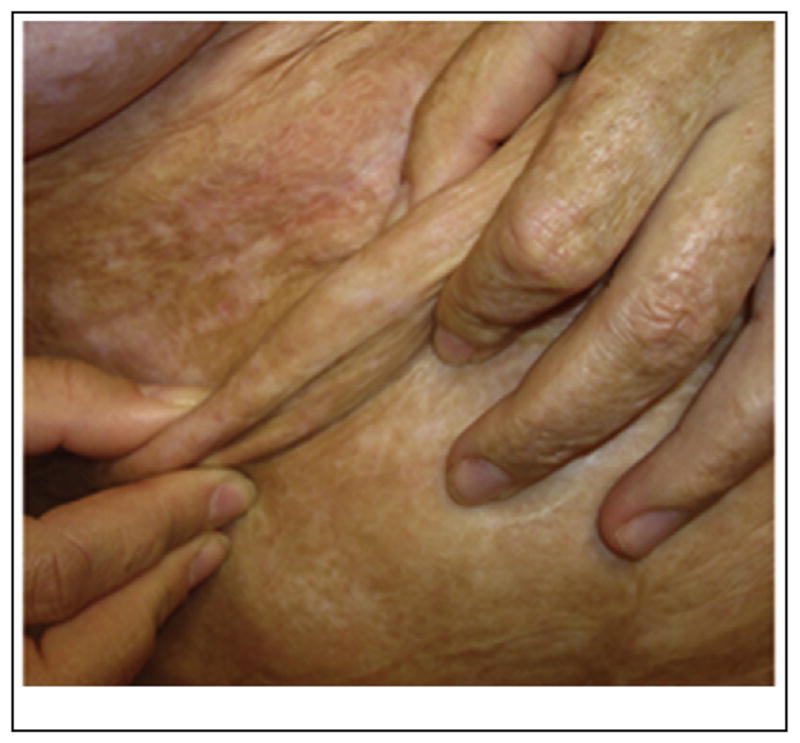
Regenerated skin in the abdomen of a female The patient had been deeply burned in the abdominal area which became scarred and lost its compliance. She was treated surgically with excision of the scar to its full depth, followed by grafting with the commercial version (Integra™) of the dermis regeneration template (DRT). Newly regenerated, compliant skin replaced the scarred area. The photo shows the regenerated skin 6 years after the initial surgery (Photo courtesy E. Dantzer, MD, France).

In contrast, grafting of wounds in skin and peripheral nerves (PN) with a collagen-based scaffold, the dermis regeneration template (DRT), has induced regeneration of these organs reliably over several years. An early version of the rationale for treatment of skin loss was reported in 1980 [1]. Results from the first clinical study with 10 massively burned patients were reported soon after [2]. DRT was physicochemically characterized as a natural polymeric network that induces *de novo* synthesis of the dermis, the key tissue in skin that fails to regenerate spontaneously [3]. Seeding of DRT with keratinocytes led to simultaneous regeneration of dermis and epidermis [3]. Even though outcomes were imperfect in these early efforts (e.g., hair and sweat glands were missing), this treatment for extensive skin loss has seen increasing use in the clinic. Increased perfection of outcome, including regeneration of hair follicles and sweat glands, was reported in subsequent studies [4]. Over 340 clinical cases of DRT use are cited in http://www.ncbi.nlm.nih.gov/pubmed/?term=Integra+substitute+skin. An important clinical advantage of induced regeneration has been the absence of morbidity that typically accompanies the replacement of organs by transplantation and other procedures. An example of a clinical result using the commercial version of DRT appears in Fig. 1.

This treatment was later extended to regeneration of peripheral nerves (PN) across long gaps between stumps resulting from transection in animals [5,6]. The relatively recent (2012–2017) elucidation of the regeneration mechanism induced by DRT both at the organ scale and the molecular scale [7–9], summarized below, provides strong motivation for studies that extend the methodology to organs other than skin and PN.

In this review we summarize the salient features of induced regeneration of skin and peripheral nerves as currently understood. A dominant element of this approach stems from the realization that a carefully standardized wound in the injured or diseased organ, together with an appropriate scaffold, provides almost everything that is required to regenerate skin and peripheral nerves.

## Why use a wound as a bioreactor to regenerate organs?

Experimental studies of regeneration of skin and PN with animals, as well as in clinical studies, have been based on use of a wound as the site for grafting a collagen-based scaffold. In clinical practice such wounds have typically resulted from accidental trauma. Increasingly, however, surgical procedures are currently being developed, designed to *generate* a wound in the intact organ that is later grafted with DRT. This elective procedure has been used to regenerate the diseased or congenitally abnormal organ, rather than an organ that has been traumatically devastated [10–13]. This development potentially increases the range for future use of a regenerative treatment to terminally diseased organs.

Although a great deal is known about the biochemistry of normal wound healing, many critical details of cell signaling events inside a wound are not yet known and cannot therefore be rationally manipulated to achieve desired clinical objectives, such as acceleration of healing or regeneration. The discovery of a regeneratively active scaffold (DRT) helps to bypass such uncertainty by inducing a benign but decisive modification of the normal wound healing process. DRT short-circuits the natural cell signaling processes during wound healing and results in an important modification of natural healing that yields physiologic tissue rather than scar. This is a result of major medical interest.

An experimental wound suitable for study of induced regeneration not only yields reproducible results from one animal to the next but also provides an accurate answer concerning the incidence or absence of a regenerative outcome. Among different types of tissue in organs [14] the stroma (connective tissue) is the singular tissue which does not regenerate spontaneously following severe injury. It follows that the most important characteristic of an experimental wound that is suitable for a screening study is that it is scrupulously free of stroma [15]. Examples of such wounds are the full-thickness skin wound, grafted with a sheet of the experimental biomaterial; and the completely transected peripheral nerve treated with the two nerve stumps placed inside a tube fabricated from the material being screened for its potential ability to regenerate [15].

The end state of the wound healing process is wound closure, a very useful reference state for organizing information that applies directly to regeneration. Wounds in skin and PN close by a combination of three processes: contraction of wound edges, scar formation and regeneration. Each of these processes of wound closure has been studied quantitatively by several authors [15].

Scar formation in skin wounds appears in the form of collagen fibers that are highly oriented in the plane of the skin wound [16–20]. In PN, collagen fibers of neural scar are arranged circumferentially around each stump [6,21]. Regenerated tissues in skin [22,23] and PN [5,6] have been definitively distinguished from scar. The macroscopic contraction force to close a wound in the rodent skin has been measured at 0.1 N [24]. The contraction force is generated by contractile cells (myofibroblasts, MFB) [25,26] that have themselves been oriented in the plane of skin by the contractile forces. Collagen fibers are synthesized by previously aligned cells, that include undifferentiated and differentiated fibroblasts (MFB). There is evidence [27] that collagen fibers synthesized by fibroblasts are deposited outside the synthesizing cell with the fiber axis in parallel to the long cell axis. The combined evidence shows that the mechanical field during wound contraction orients the MFB which in turn synthesize the oriented fibers characteristic of scar [9].

In a transected peripheral nerve, MFB have been shown to be arranged circumferentially around each stump in the form of a capsule [6]. Quantitative evidence shows that the diameter of nerve stumps is steadily reduced as the thickness of the contractile cell capsule increases [6]. It has been concluded that nerve stumps surrounded by MFB are subjected to circumferential compression of the cross-section area, which also explains the observed substantial reduction in number of myelinated fibers [8,9].

The three processes that are responsible for wound closure are related closely. Blocking of wound contraction by DRT, a well-known contraction blocker [28], abolishes the orientation of collagen fibers both in sin wounds and PN wounds. In these DRT-treated wounds the morphology of scar was eliminated [9]. Instead, the outcome of the healing process was either the random morphology of dermis (skin wounds) or the formation of nerves with large, almost undeformed, diameter and a near normal number of myelinated axons. It follows from the above that scar formation is entirely dependent on contraction in normally healing (untreated) wounds [9].

Additional evidence [9,15] suggests that wound contraction must be blocked in order to close wounds by regeneration. For example, there is evidence from studies of spontaneous wound healing in a number of species (e.g., axolotl skin, rabbit ear, human oral mucosa) that wound contraction and regeneration do not occur together in the same wound model [9,15]. Further, when contraction-blocking DRT is used to graft wounds, regeneration is observed both in skin and PN wounds. Although not signifying a causal relation, these observations suggest relatively clearly that wound contraction and regeneration are antagonistic processes during closure of adult mammalian wounds [3,7,9,15].

Following this brief excursion into wound healing and wound closure we now look closely at the polymeric material that is responsible for blocking contraction and simultaneously inducing regeneration.

## The surface chemistry of a collagen scaffold

Collagen is a natural polymer with a complex but largely described structure [29,30]. Collagen microfibrils (pentamers) comprise collagen molecules arranged in groups of five that become organized into thin fibrils and thicker fibers, ranging in diameter between 25 and 400 nm. Collagen fibrils belong to a higher structural order than the pentamer [29]; these are characterized by a semi-crystalline structure identified by a 67-nm periodicity, commonly referred to as the collagen banding pattern. Type I collagen from a variety of animal tissues has been purified and further processed for use as a biomaterial or for *in vitro* assays [7,29,30].

Of its many structural features, the surface chemistry of collagen fibers currently appears to be the most critical variable in induction of regeneration. This finding expands greatly the customary view of collagen as a fibrous polymeric material that simply lends stiffness and strength to animal tissues. The reason why the surface chemistry of collagen assumes such a position of importance in studies of regeneration is the participation of collagen fibers in extensive and critical communication processes between cells and the extracellular matrix (ECM) [31], that appear to govern induction of regeneration of skin and PN. In particular, it appears that contractile cells and the collagen surface engage in a specific binding interaction that blocks the wound contraction process.

Located on the surface of collagen fibers are several adhesion ligands, small peptide motifs that bind specifically to cell integrins. For example, the hexapeptide motif GFOGER (glycine-phenylalanine-hydroxyproline-glycine-glutamic acid-arginine), corresponds to residues 502–516 of the alpha(1)(I) chain of collagen. This motif, as well as the GLOGER hexapeptide, were both identified as high affinity binding sites both for α_1_β_1_ and α_2_β_1_ integrins in type I collagen fibers [32,33].

We now consider the other side of the cell-ECM binding interaction, namely, cell receptors which provide the cell with a perception of its microenvironment. An important group of receptors, which bind to ECM proteins, including collagen, are members of the integrin family [31]. Binding of cells to collagen via their integrins typically mediates changes in cell phenotype (biological behavior). Wound contraction processes mediated by adhesion of α_1_β_1_ and α_2_β_1_ to collagen have been widely reported. As one of many examples, myofibroblast differentiation, identified by expression of alpha smooth muscle actin (αSMA), was induced by α_1_β_1_ [34,35].

Quantitative detection of the specific binding interaction between integrin receptors in contractile cells and adhesion ligands in the collagen surface has been made using multi-photon microscopy [8]. Very briefly described, fluorescently-labelled markers of integrins were prepared following isolation and purification of chain fragments (referred to as the I-domains) of integrin molecules a1b1 and a2b1 that normally bind to ligands on collagen. These markers have binding properties that appear to be identical to those of the adhesion receptors and can be used to identify the location of adhesion ligands on the surface of the collagen scaffold. This new methodology was used to measure the density of different ligands for integrins α_1_β_1_ and α_2_β_1_ on collagen scaffolds which differed in ability to induce regeneration of peripheral nerves in animals but were otherwise similar in structure [8]. Although the data are incomplete they are nevertheless consistent with a mechanism of scaffold activity that depends on cell adhesion on the collagen surface, mediated by high levels of ligand densities for the two integrins. The detailed methodology has been described [8,36].

## How wound contraction is blocked by a collagen scaffold with highly specific structure

We still have not answered an important question: How is the contractile machinery of skin and PN wounds affected following binding to the contraction-blocking DRT scaffold?

**Fig. 2:**
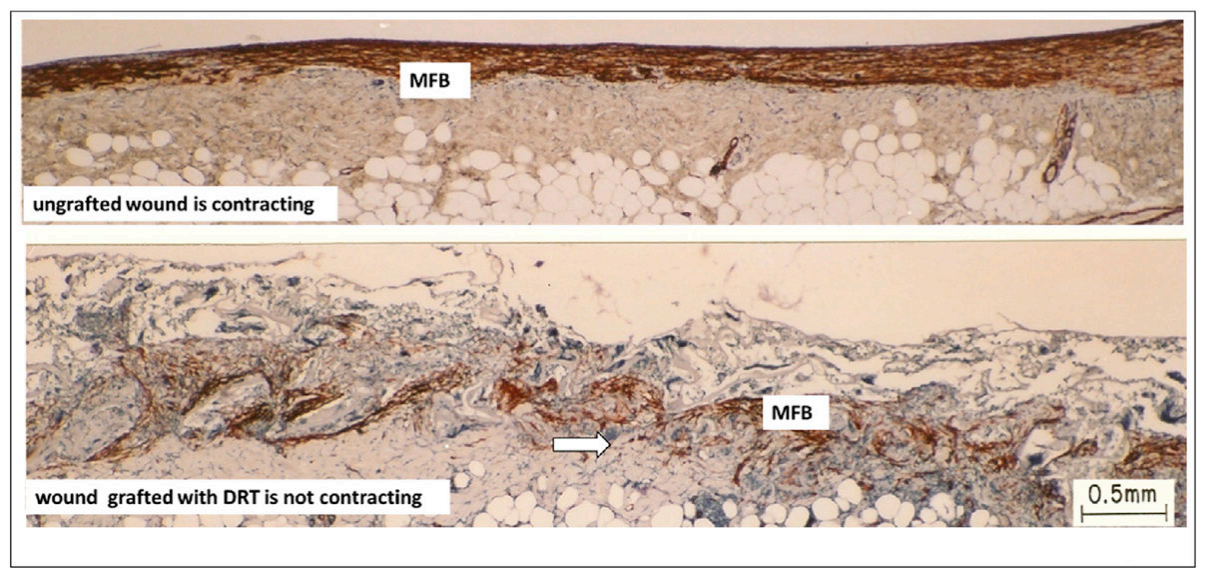
DRT changes the phenotype of contractile cells and cancels wound contraction Sharply contrasting views of two full-thickness skin wounds (guinea pig dorsum). Contractile cells (myofibroblasts, MFB, red brown) were stained with an antibody to alpha smooth muscle actin. Top: Untreated skin wound (no DRT) is contracting vigorously on day 10. MFB form dense assemblies comprised of cells with their long axes oriented along the direction of wound contraction. Bottom: Wound grafted with DRT is not contracting on day 11. MFB are much fewer, their assemblies have largely dispersed and their long axes are not oriented along the direction of wound contraction. Arrows: scaffold struts (photos by K. Troxel; adapted from [9]).

**Fig. 3:**
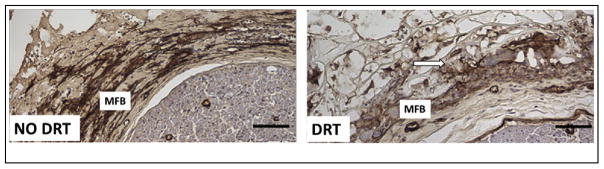
Scaffold disorganizes contractile cells in peripheral nerve wounds Two peripheral nerve wounds observed 14 days after transection of the rat sciatic nerve. The top-left quarter of the nerve stump cross section (neural tissue) is shown at bottom right in each photo. Immunohistochemical localization of α-smooth muscle actin (characteristic of myofibroblasts, MFB) is observed in the contractile cell capsule surrounding the stumps (brown). Left: Untreated wound (no DRT). MFB are dense, assembled closely, with their long axes oriented circumferentially around the neural tissue. MFB apply circumferential compressive forces that shrink the nerve diameter and yield a poorly regenerated nerve. Right: Nerve stumps were inserted in a tube fabricated from DRT. MFB show lower density, dispersed MFB assemblies and lack of circumferential orientation of MFB axes (right) compared to untreated nerve (left), These changes represent a dramatic loss of contractile MFB phenotype. Contraction of the nerve stump diameter is blocked in the wound treated with DRT, leading to a regenerated nerve of high quality. Scale bars: 100 μm (photos by E. Soller; adapted from [9]).

**Fig. 4:**
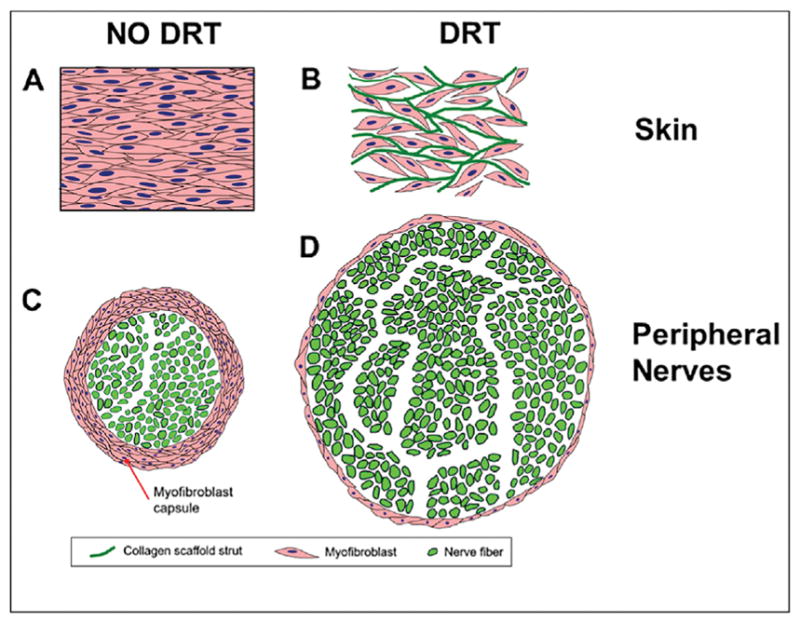
Schematic representation of contraction blocking by the DRT scaffold in skin and nerves that leads to scarless healing (regeneration) (A) In untreated skin wounds the normal mechanical force of wound contraction orients the long axes of myofibroblasts (MFB; pink) along a major contraction axis. (B) In the presence of DRT, MFB adhere on the DRT surface via integrin-ligand interactions. MFB become disassembled and distributed inside the pores of the scaffold. (C) In an untreated peripheral nerve wound, a thick capsule of contractile cells (pink) is arranged circumferentially around the nerve stump, compressing the stump diameter and preventing stump reconnection. (D) In the presence of DRT, the contractile cell capsule is very thin, the stump diameter is large, eventually facilitating stump reconnection (graphic by DS Tzeranis; adapted from [9]).

Obvious changes in MFB morphology both in the presence and absence of DRT provide an answer to this question. Comparing photos of immunohistochemically stained tissue sections in skin wounds (Fig. 2) and nerve wounds (Fig. 3) we observe that, in the presence of DRT, a) MFB assemblies have become dispersed, b) the long axes of MFB have lost their alignment and become more or less randomly oriented in space, and c) the number of MFB has become much lower. The combination of these three morphological changes appears to explain, qualitatively at least, the fact that wounds do not contract in the presence of DRT. This fact describes a rare example of a major change in cell phenotype brought about by a biomaterial. Furthermore, the combined evidence suggests strongly that skin and PN wounds heal by a very similar mechanism: a) wound healing in both untreated organs is largely controlled by wound contraction and b) DRT effectively blocks contraction in both. While this result was well known about skin wounds, it is a recent finding for PN wounds. A schematic representation of the effect of DRT in skin wounds and PN wounds is shown in Fig. 4.

To date, three structural features of DRT have been shown to be necessary for the phenotype change: The average pore size must lie between about 20 and 125 microns; the degradation half-life must take values near 14 ± 7 days; and the surface of the scaffold has to include a substantial number of ligands that interact with integrins α_1_β_1_ and α_2_β_1_ [7]. Future studies will focus on the minimally required density of ligands. It is important to note that several related collagen scaffolds with structures that deviated from the rules presented here were found to be largely or totally inactive.

A plausible pathway for contraction blocking at the cell and tissue scale has emerged. In this hypothetical pathway, a MFB migrates into a scaffold through pores that are large enough for it (notice requirement for lower limit in pore size) but not so large that the specific surface, which decreases regularly with increase in pore size in any porous material, becomes inadequate to bind all cells in the wound (upper limit in pore size). The scaffold half-life has to be adjusted to a level that ensures the simultaneous presence of MFB and a fragment of scaffold surface sufficient for cell adhesion. Considering that MFB are absent from the wound in the first few days following injury, and that they suffer apoptosis after about 30 days, the observed scaffold half-life of about 14 days is reasonable. Lastly, the presence on the scaffold surface of ligands capable of binding integrins of contractile cells appears to be required by a scenario which calls for such binding as a prerequisite to the phenotype change.

## Future outlook of regeneration treatments in medicine

Extension of this regenerative treatment to clinical use appears to have been limited so far to skin and peripheral nerves. Consider, however, that untreated wounds both in skin and peripheral nerves close principally by contraction; and that, in both organs, treatment of wounds with DRT cancels contraction and leads to regeneration. These facts suggest strongly that the DRT methodology could be extended to other organs which respond to injury by wound contraction. Examples of organs which are considered candidates for future use of this paradigm are the mouse liver, which contracts and forms scar following laceration [37]; the rat spinal cord, which forms scar and shows presence of contractile cells following complete transection [38]; and the rabbit conjunctiva, which contracts and forms scar following full-thickness removal of conjunctival tissue and Tenon’s capsule [39].

We note that regeneration by use of DRT does not require that the wound be inflicted accidentally, as in burns, lacerated nerves, or other kinds of severe trauma. A number of clinical studies have been reported in which nontraumatic diseases in skin that have resulted in cosmetic defects [10–13] have been treated successfully by this regeneration approach. In these cases, an elective surgical procedure of excision of a congenitally malformed or diseased organ, was followed by grafting with DRT, a process that led to regeneration of physiologic skin at the excised site. These clinical successes suggest strongly the future applicability of induced regeneration to appropriately selected organs that are terminally diseased, not necessarily injured, and are candidates for organ transplantation.

## Abbreviations

DRT: dermis regeneration template
MFB: myofibroblasts
GFOGER: glycine-phenylalanine-hydroxyproline-glycine-glutamic acid-arginine
GLOGER: glycine-leucine-hydroxyproline-glycine-glutamic acid-argi-nine
PN: peripheral nerve
αSMA: alpha smooth muscle actin
